# Perceptual Decision Making “Through the Eyes” of a Large-Scale Neural Model of V1

**DOI:** 10.3389/fpsyg.2013.00161

**Published:** 2013-04-19

**Authors:** Jianing V. Shi, Jim Wielaard, R. Theodore Smith, Paul Sajda

**Affiliations:** ^1^Department of Biomedical Engineering, Columbia UniversityNew York, NY, USA; ^2^Department of Ophthalmology, New York Presbyterian HospitalNew York, NY, USA

**Keywords:** computational modeling, decision making, neuronal network, sparse coding

## Abstract

Sparse coding has been posited as an efficient information processing strategy employed by sensory systems, particularly visual cortex. Substantial theoretical and experimental work has focused on the issue of sparse encoding, namely how the early visual system maps the scene into a sparse representation. In this paper we investigate the complementary issue of sparse decoding, for example given activity generated by a realistic mapping of the visual scene to neuronal spike trains, how do downstream neurons best utilize this representation to generate a “decision.” Specifically we consider both sparse (L1-regularized) and non-sparse (L2 regularized) linear decoding for mapping the neural dynamics of a large-scale spiking neuron model of primary visual cortex (V1) to a two alternative forced choice (2-AFC) perceptual decision. We show that while both sparse and non-sparse linear decoding yield discrimination results quantitatively consistent with human psychophysics, sparse linear decoding is more efficient in terms of the number of selected informative dimension.

## Introduction

How the brain represents information and how such a representation is ultimately utilized to form decisions and mediate behavior are fundamental questions in systems neuroscience, being addressed at many different scales from single-unit recordings to neuroimaging across the brain. Work in theoretical neuroscience has argued that a useful information processing strategy for the brain is to map sensory information into a sparse representation – the sparse coding hypothesis (Olshausen and Field, 1996a). Sparse coding has been viewed as an optimal strategy for minimizing redundancy and has been experimentally observed and theoretically justified for a number of sensory systems including visual (Baddeley, 1996; Dan et al., 1996; Baddeley et al., 1997; Vinje and Gallant, 2000, 2002), auditory (Hahnloser et al., 2002; Hromadka et al., 2008; Greene et al., 2009), olfactory (Perez-Orive et al., 2002; Szyszka et al., 2005; Rinberg et al., 2006), and motor (Brecht et al., 2004) systems. It is also considered efficient from a metabolic and energy perspective.

Sparse coding has received substantial attention in the visual neurosciences (Baddeley, 1996; Dan et al., 1996; Olshausen and Field, 1996a,b, 1997, 2004; Rolls and Tovee, 1995; Vinje and Gallant, 2000, 2002; Simoncelli and Olshausen, 2001), particularly from the perspective of encoding. For example, it has been shown that imposing sparsity constraints on rate-based neuronal models yields receptive fields that resemble those of simple cells in V1 (Olshausen and Field, 1996a). Others have shown how sparse encoding can emerge temporally and is consistent with the spike time distributions of visual neurons (Baddeley, 1996). Some have looked more closely at the specific nature and degree of the sparse encoding, investigating differences between “soft” and “hard” sparseness constraints (Rehn and Sommer, 2007). Many conclude that the visual system maps the visual scene into a sparse representation. However given such a sparse encoding strategy, how does the rest of the visual system utilize it for robust and efficient object recognition? How is the sparse representation exploited downstream to yield the behavior we observe?

In this paper we consider the problem from the perspective of decoding activity from a large neuronal population (>1000s neurons) simulated via a physiologically realistic model of primary visual cortex. Related recent experimental work has investigated linear decoding for orientation discrimination by decoding neural activity from approximately 100 neurons in macaque V1 (Graf et al., 2011). The reason for considering such a modeling approach is that a mesoscopic analysis of 1000s of neurons is not possible given the current state-of-the-art electrophysiology, since experimentalists are still not able to record, in awake and behaving animals, thousands of neurons at millisecond timescales.

Specifically we investigate how a representation of the visual scene, constructed by early visual processing, is best linearly decoded and mapped to behavior within a 2-AFC perceptual decision paradigm. We investigate sparse linear decoding not only in terms of discrimination accuracy, but also as an avenue for examining the informative dimensions within the neural activities. We compare the discriminatory predictions generated by the decoding scheme to human psychophysics. Our approach enables us to analyze the role of sparse coding in large neuronal populations in relation to decoding accuracy and efficiency.

## Materials and Methods

### Perceptual decision making paradigm

We adapted a 2-AFC paradigm of face and car discrimination, where a set of 12 face (Max Plank Institute face database) and 12 car grayscale images were used. The car image database was the same used in Philiastides and Sajda (2006) and Philiastides et al. (2006), which was constructed by taking images from the internet, segmenting the car from the background, converting the image to grayscale, and then resizing to be comparable as the face images. The pose of the faces and cars was also matched across the entire database and was sampled at random (left, right, center) for the training and test cases. All the images (512 × 512 pixels, 8 bits/pixel) were equated for spatial frequency, luminance, and contrast. The phase spectra of the images were manipulated using the weighted mean phase method (Dakin et al., 2002) to introduce noise, resulting in a set of images graded by phase coherence. Specifically we computed the 2D Fourier transform of each image, and constructed the average magnitude spectra by averaging across all images. The phase spectra of an image were constructed by computing a weighted sum of the phase spectra of the original image (ϕ_image_) and that of random noise (ϕ_noise_).

**Figure 1:**
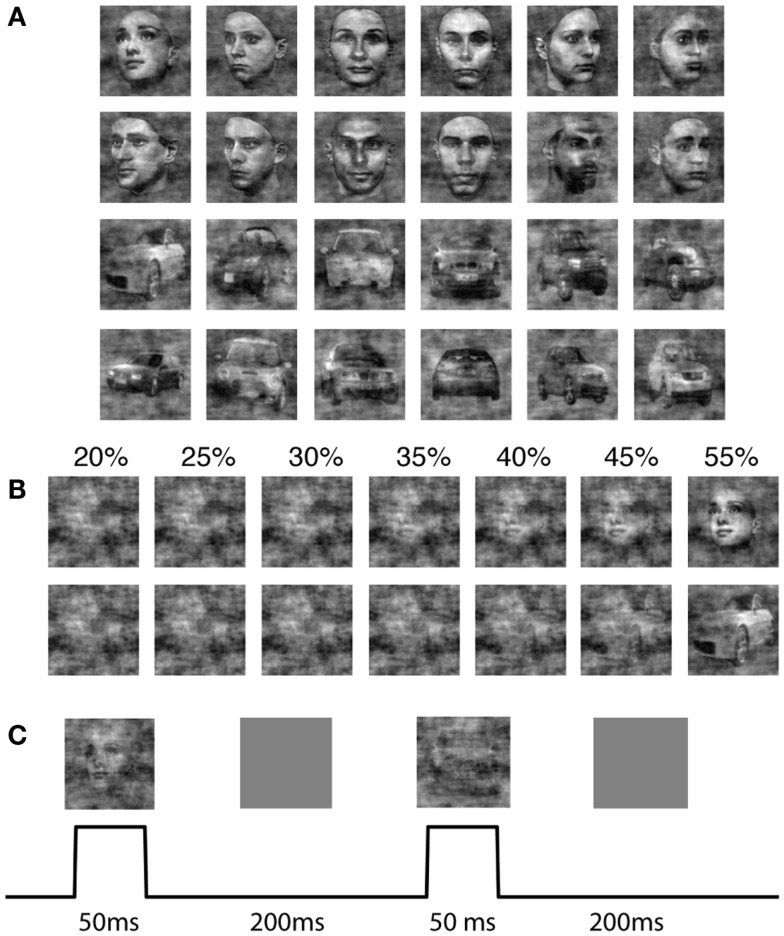
**The stimulus set for the 2-AFC perceptual decision making task**. **(A)** Shown are 12 face and 12 car images at phase coherence 55%. **(B)** One sample face and one sample car image, at phase coherences varying from 20 to 55%. **(C)** Design and timing of the simulated psychophysics experiment for the model.

Each image subtended 2° × 2° of visual angle, and the background screen was set to a mean luminance gray. The image size was set to match the size of the V1 model, which covered 4 mm^2^ of cortical sheet. Figure 1 shows examples of the face and car images used in the experiment as well as the effect on the discriminability of the image class when varying the phase coherence.

The sequence of images was input to the model where an image was flashed for 50 ms, followed by a gray mean luminance image with an inter-stimulus-interval (ISI) of 200 ms (Figure 1C). Since simulating the model is computationally expensive, we minimized the simulation time by choosing an ISI which was as small as possible yet did not result in network dynamics leaking across trials. We conducted pilot experiments that showed that network activity settled to background levels approximately 200 ms after stimulus offset. We ran the simulation for each of the two classes, face and car, at different coherence levels (20, 25, 30, 35, 40, 45, 55%) respectively. Each image was repeated by 30 trials in the simulation, where the sequence of trials was randomly generated. In each simulation, we randomized the order of different images, making sure not to push the model into a periodic response pattern.

Parallel to simulating the model response, we conducted human psychophysics experiments. Ten volunteer subjects were recruited. All participants provided written informed consent, as approved by the Columbia University Institutional Review Board. All the subjects were healthy with corrected visual acuity of 20/20. Psychophysics testing was administered in a monocular manner. Images of different phase coherences were randomized in the psychophysics experiment. During the experiment subjects were instructed to fixate at the center of the images, and to make a decision on whether they saw a face or car, as soon as possible, by pressing one of two buttons with their right hand. The ISI for human psychophysics experiments was longer and randomized between 2500 and 3000 ms in order to provide for a comfortable reaction time and to reduce the subjects’ ability to predict the time of the next image. A Dell computer with an nVIDIA GeForce4 MX 440 AGP8X graphics card and E-Prime software controlled the stimulus presentation.

### Model summary

**Figure 2:**
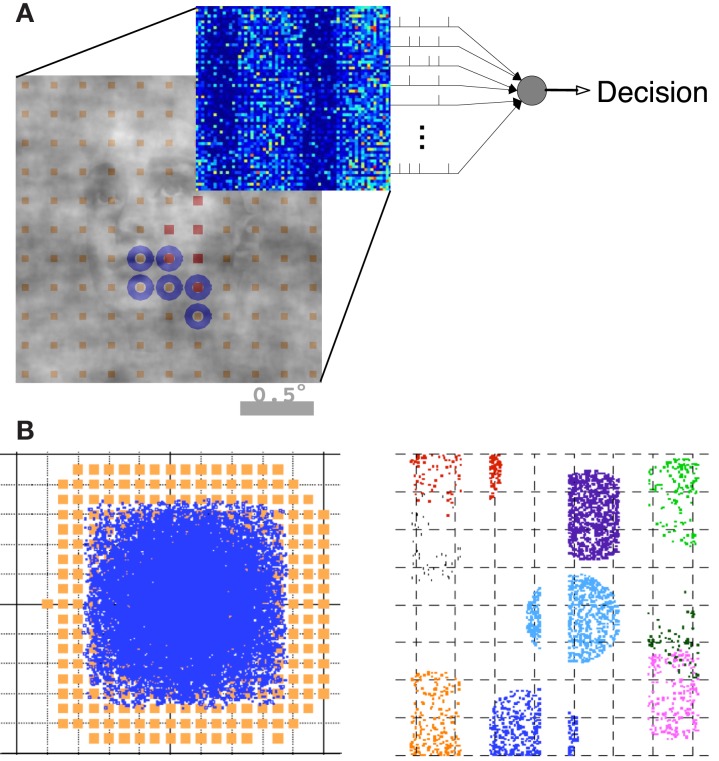
**Summary of the model architecture**. **(A)** The model is comprises of the encoding and decoding components. **(B)** Architecture of the V1 model, where receptive fields and LGN axon targets are viewed in the visual space (left) and cortical space (right). Details can be found in Wielaard and Sajda (2006a).

An overview of the model architecture and decoding is illustrated in Figure 2. We modeled the early visual pathway with a feedforward lateral geniculate nucleus (LGN) input and a recurrent spiking neuron network of the input layers of (4Cα/β) of primary visual cortex (V1). We model the short-range connectivity within the V1 layer, without feedback from higher areas. We simulated a magnocellular version of the model, the details of which have been described previously (Wielaard and Sajda, 2006a,b, 2007). Note our model is a variant of an earlier V1 model (McLaughlin et al., 2000; Wielaard et al., 2001).

In brief, the model consists of a layer of *N* (4096) conductance-based integrate-and-fire point neurons (one compartment), representing about a 2 × 2 mm^2^ piece of a V1 input layer (layer 4C). Our model of V1 consists of 75% excitatory neurons and 25% inhibitory neurons. In the model, 30% of both the excitatory and inhibitory cell populations receive LGN input. In agreement with experimental findings, the LGN neurons are modeled as rectified center-surround linear spatio-temporal filters. Sizes for center and surround were taken from experimental data (Hicks et al., 1983; Derrington and Lennie, 1984; Shapley, 1990; Spear et al., 1994; Croner and Kaplan, 1995; Benardete and Kaplan, 1999). Noise, cortical interactions, and LGN input are assumed to act additively in contributing to the total conductance of a cell. The noise term is modeled as Poisson spike train convolved with a kernel which comprises a fast AMPA component and a slow NMDA component (see Supplementary Materials in Wielaard and Sajda, 2006a).

The LGN RF centers were organized on a square lattice. These lattice spacing and consequent LGN receptive field densities imply LGN cellular magnification factors that are in the range of the experimental data available for macaque (Malpeli et al., 1996). The connection structure between LGN cells and cortical cells is made so as to establish ocular dominance bands and a slight orientation preference which is organized in pinwheels (Blasdel, 1992). It is further constructed under the constraint that the LGN axonal arbor sizes in V1 do not exceed the anatomically established values of 1.2 mm (Blasdel and Lund, 1983; Freund et al., 1989).

In the construction of the model our objective was to keep the parameters deterministic and uniform as much as possible. This enhances the transparency of the model, while at the same time provides insight into what factors may be essential for the considerable diversity observed in the responses of V1 cells.

### Sparse decoding

We used a linear decoder to map the spatio-temporal activity in the V1 model to a decision on whether the input stimulus is a face or a car. We employed a sparsity constraint on the decoder in order to control the dimension of the effective feature space. Sparse decoding has been previously investigated for decoding real electrophysiological data, for instance by Chen et al. (2006), Palmer et al. (2007), and Quiroga et al. (2007).

Since a primary purpose of using the decoder is to identify informative dimensions in the neurodynamics, we estimate new decoder parameters at each stimulus noise level (coherence level) independently. Alternatively we could train a decoder at the highest coherence level and test the decoder at each coherence level. In this paper we focus on the first approach, since we view our decoder as a tool for analyzing the information content in the neurodynamics and how downstream neurons might best decode this information for discrimination.

We constructed an optimal decoder to read out the information in our spike neuron model, fully exploring the spatio-temporal dynamics. The spike train for each neuron in the population is $s_{i,k}(t)=\sum_{l}\text{δ(}t-t_{i,k,l}\text{)}$, where *t* ∈ [0,250] ms, *i* = 1… *N* is the index for neurons, *k* = 1… *M* is the index for trials, *l* = 1… *P* is the index for spikes. Based on the population spike trains, we estimated the firing rate on each trial by counting the number of spikes within a time bin of width τ, resulting in a spike count matrix $r_{i,j,k}=\int_{\text{(}j-1\text{)}\tau +1}^{j\text{τ}}s_{i,k}(t)\text{dt}$, where *i* = 1… *N* represents the *i*th neuron, *j* = 1… *T*/τ represents the *j*th time bin, *k* = 1… *M* represents the *k*th trial. Note that we explored decoding using time bins of different length. When τ = 25 ms, we assume that information is encoded in both neuron and time, since the firing rate is closer to instantaneous firing rate; when τ = 250 ms, we integrate the spiking activity over the entire trial, leading to a rate-based representation of information. A separate *post hoc* analysis showed that 25 ms was in fact the bin width that yielded the highest discrimination accuracy (bin width varied from 5 to 250 ms). The class label of each sample *b_k_* takes the value of {-1, + 1} representing either face or car with *M* being the number of trials. In order to explore the information within the spatio-temporal dynamics, we compute the weighted sum of firing rate over different neurons and time bins. This leads to seeking the solution of the following constrained minimization problem,

(1) $$\left\{w,v\right\}=\underset{w,v}{\mathrm{argmin}}\frac{1}{M}\overset{M}{\underset{k=1}{\sum }}L\left(\left(w^{T}x_{k}+v\right)b_{k}\right)+\lambda J\left(w\right),$$

where the first term is the empirical logistic loss function, and the second term is the regularization function, with λ > 0 as the regularization parameter. We create a stacked version of the spike count matrix; *x_l,k_* = *r_i,*j*,*k*_* with *l* = (*i* − 1)*N* + *j*, i.e., stacking the neuron and time bin dimensions together. The resulting linear decoder can be geometrically interpreted as a hyperplane that separates the classes of face and car, where *w* represents the weights for the linear decoder, and *v* is the offset. In the case of the sparse decoder, we use an L1 regularization term $J(w)={\left\|w\right\|}_{1}$; alternatively for the non-sparse decoder, we use the L2 regularization $J(w)={\left\|w\right\|}_{2}^{2}$. In the language of Bayesian analysis, the logistic loss term comes from maximum likelihood, L1 corresponds to the Laplacian prior, and L2 corresponds to the Gaussian prior. L1-regularized logistic regression results in a sparse solution for the weights (Krishnapuram et al., 2005; Koh et al., 2007; Meier et al., 2008). So-called “sparse logistic regression” serves as an approach for feature selection, where features that are most informative about the classification survive in the form of non-zero weights (Ng, 2004). We developed an efficient and accurate method to solve this optimization problem (Shi et al., 2010, 2011). Once we learn the hyperplane, for any new image, we can predict the image category via the sign of $w^{T}x_{k}+v$.

**Figure 3:**
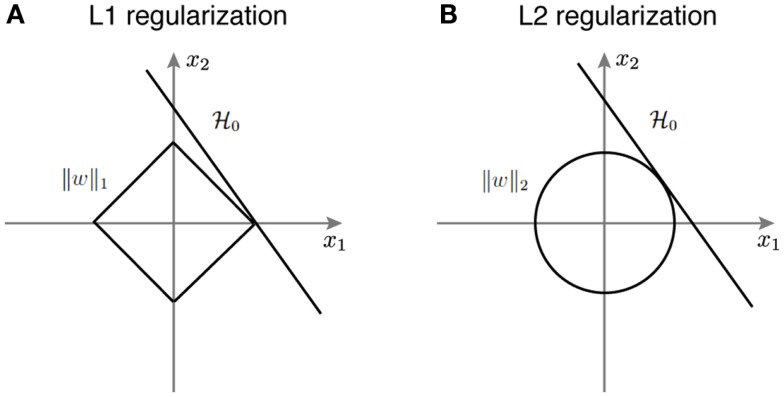
**A schematic illustration of how different regularization terms lead to sparse and non-sparse solutions in the linear classifier**. **(A)** L1 regularization corresponds to the diamond shaped ball centered around the origin. **(B)** L2 regularization corresponds to the spherical ball centered around the origin.

Figure 3 provides a geometric intuition of why L1 and L2 regularization lead to sparse and non-sparse solutions, respectively. The solution of L1 or L2 regularized logistic regression is the intersection of the regularization geometry and a hyperplane. Figure 3A shows the L1 regularization corresponds to the diamond shaped ball centered at the origin. As one increases the regularization parameter λ, the L1 ball grows and the solution is the point when it hits the hyperplane. Given the geometry of L1 ball, the solution is more likely to be sparse. Figure 3B shows the L2 regularized logistic regression, where the geometry of the L2 ball is a sphere, therefore leading to a non-sparse solution.

### Cross validation

Training and testing were carried out on different sets of images, each containing six face images and six car images, with 30 trials per image. Tenfold cross validation was used on the training set, while the final weights applied on the testing set are estimated using Jackknife estimation to reduce the bias. A regularization path was also employed, where a family of λ’s is used. Given that different values of λ offer different levels of sparsity, we chose λ that maximizes discrimination accuracy on the training dataset after cross validation. We used this hyperparameter on the testing dataset to calculate the final discrimination accuracy. In order to identify the time windows that are critical for reading out information in the V1 model, we used two approaches. One way to utilize dynamics was based on a heuristic approach, where we only consider dynamics during *t* ∈ [50, 150] ms, given that the V1 model has a delay of 50 ms after stimulus onset and the length of activation is about 100 ms. In a second approach, we optimized the temporal window by an adaptive technique, where we search for an optimal window that results in the best decoding performance. In the adaptive technique, we systematically varied the latency and width of the window, and computed the corresponding Az (area under ROC curve) values through cross validation. The best window is the one that results in the highest Az value.

### Measuring sparseness

We characterize the sparseness of the neural representation in the population spike trains, for both the temporal and spatial domains. According to Willmore and Tolhurst (2001), lifetime sparseness describes the activity of a single neuron over time, while population sparseness characterizes the activity of a population of neurons for a given time window. We estimate instantaneous firing rates using a Gaussian window 25 ms wide with a standard deviation of 5 ms. Sparseness in firing rates can be measured by kurtosis (Olshausen and Field, 2004), namely the forth moment relative to the variance squared.

Using the sparse decoding framework, we are able to identify the informative dimensions that are critical for our specific decision making task. We define “informative dimensions” as the number of non-zero weights in the decoder, which is equal to the cardinality of the weight vector. Informative dimensions thus reflect the number of non-zeros in the spatio-temporal “word.” Note one neuron can be selected by the decoder at multiple time bins, therefore, we define “informative neurons” as the number of neurons having at least one non-zero weight across different time bins.

### Statistical tests

We used a likelihood ratio test to evaluate the goodness of fit. We fit a single Weibull curve jointly to both the psychometric and neurometric dataset (dof = 4), as well as fitting two Weibull curve independently to both dataset (dof = 8). We computed the likelihood ratio using $D=-2\ln\left(l_{j}∕l_{p}l_{n}\right)$. The null hypothesis is psychometric and neurometric data can be described by the same curve, and the decision rule is based on the Chi-square statistics χ^2^. If *p* > 0.05, do not reject null hypothesis; otherwise, reject null hypothesis.

## Results

### Encoding by the V1 model

**Figure 4:**
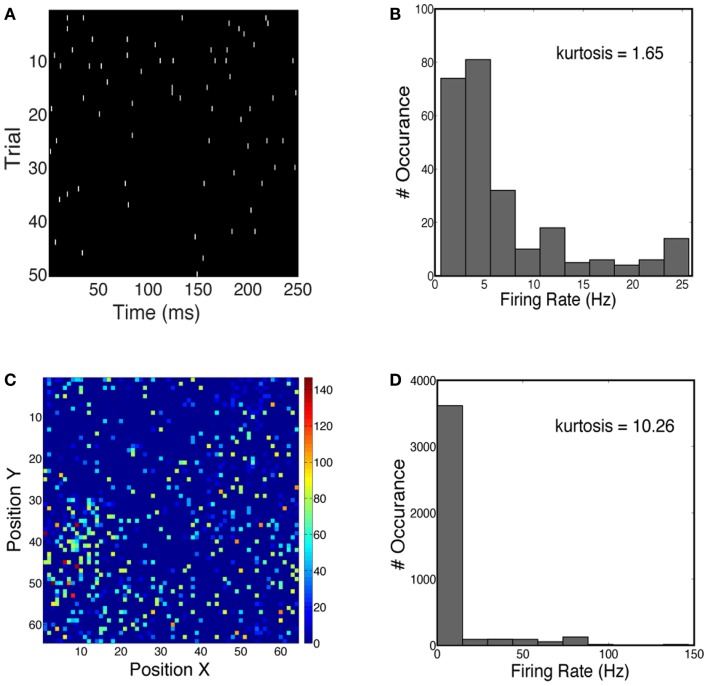
**(A)** Spike trains of one example neuron over 50 trials, simulated for a face stimulus. **(B)** Distribution of firing rates has a kurtosis of 1.65, indicative of temporal sparseness. **(C)** Spatial distribution of instantaneous firing rates over all neurons in the network. Firing rates are computed at 50 ms post-stimulus for one trial. **(D)** Distribution of firing rates has a kurtosis of 10.26.

We first characterized the sparseness in the neuronal population activity given face and car stimuli (see Materials and Methods). We measured both the lifetime sparseness and population sparseness for the network using a flashed presentation of the sample images. Figure 4A shows the time course of the firing rate for one example neuron over 50 trials. The firing rate of this neuron is sparse over time. One can also see the increase in firing rate 50–100 ms shortly after the stimulus onset. Figure 4B plots the histogram of firing rates for this neuron, averaged over 50 trials, showing that the kurtosis is 1.65, where a positive kurtosis indicates sparseness and a kurtosis of zero indicates a Gaussian distribution. Similarly, we also measured sparseness in the spatial domain. Figure 4C shows the firing rates of all neurons at 50 ms during one trial. The distribution of firing rates is shown in Figure 4D, with a kurtosis of 10.26. Thus for the stimuli we consider for constructing our 2-AFC discrimination paradigm (faces vs. cars), the V1 model produced population responses which are relatively spatially sparse and marginally temporally sparse. However they are not highly sparse (e.g., kurtosis > 100), as has been seen in models which impose hard sparseness (Rehn and Sommer, 2007).

### Decoding spatially averaged firing rate

**Figure 5:**
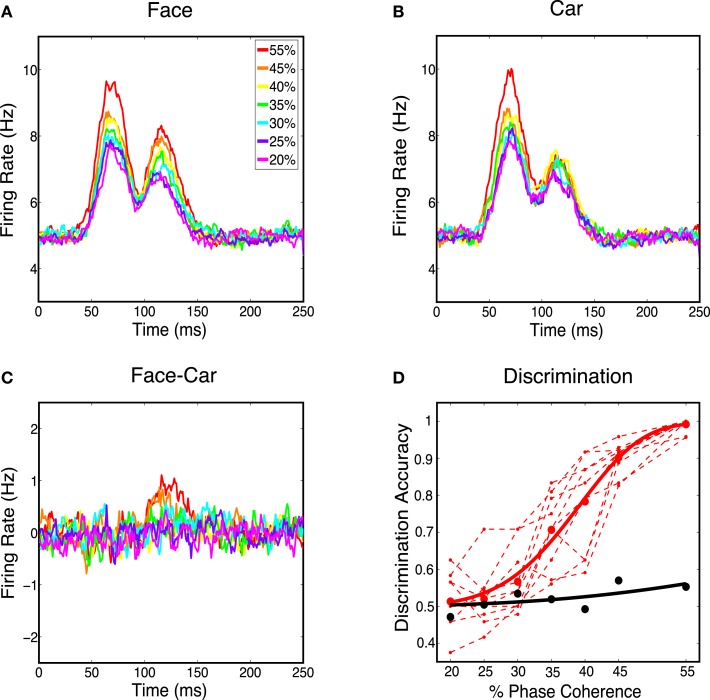
**(A)** Average response over all the model’s cortical neurons for all face stimuli. **(B)** Average response over all the Magnocortical neurons for all car stimuli. **(C)** Average response difference between face and car stimuli for the Magno system. **(D)** Neurometric curve by decoding spatially averaged firing rates for the V1 model (thick black curve) is plotted, together with the group average psychometric curve fitted across 10 subjects (thick red curve), and psychophysical performance of 10 individual subjects (thin dashed red lines).

Next we analyzed the spatially averaged firing rate from the V1 model to see whether it carried information for discriminating stimulus class. Specifically we constructed a proxy for multi-unit activity by averaging firing rates over all 4096 cortical neurons and all 30 trials for each phase coherence level. We first investigated whether spatially averaged firing rates were sensitive to phase coherence levels in the stimulus. Figure 5 shows that the spatially averaged firing rate decreases as the coherence level decreases for face trials and car trials. This is consistent with the V1 neurons being selective to oriented edge energies, which are reduced as the phase coherence decreases. Although spatially averaged firing rates in V1 have never been directly observed for this experimental paradigm, evidence for graded responses, as a function of task difficulty (i.e., coherence level), have been observed in full scalp EEG, particularly in electrodes over visual cortex (Philiastides et al., 2006).

We then considered the difference in the spatially averaged firing rate between face and car trials. Figure 5C shows a graded response for the average difference, with the trend being toward smaller differences for lower coherence levels. To investigate how discriminating this difference was on a trial-to-trial basis, we constructed histograms of the spatially averaged firing rates for both face and car trials. We constructed a neurometric function by computing the area under the receiver operating curve (ROC) curve (i.e., area given as Az) at each coherence level and fitting a Weibull function to these points. The neurometric curve was constructed based on the time window from 50–150 ms post-stimulus, which is the time window with the maximum mean difference in the spatially average firing rates for the two stimulus classes. The resulting neurometric curve (black curve), shown in Figure 5D, was a poor match to the group psychometric curve (red curve), which was computed by averaging over the behavioral data of 10 human subjects performing the same two-class discrimination task. The finding that the stimulus class, for such complex stimuli as faces and cars, cannot be decoded from the spatially averaged firing rate is also consistent with previous findings measured by EEG (Philiastides and Sajda, 2006), where EEG in visual cortex is selective for coherence level but not selective for image class.

### Decoding spatio-temporal dynamics

The above observation prompted us to investigate whether a more fine-grained mesoscopic decoding strategy holds information for stimulus class discrimination. We investigated a sparse decoding strategy that exploits the spatio-temporal neurodynamics produced by the V1 model.

**Figure 6:**
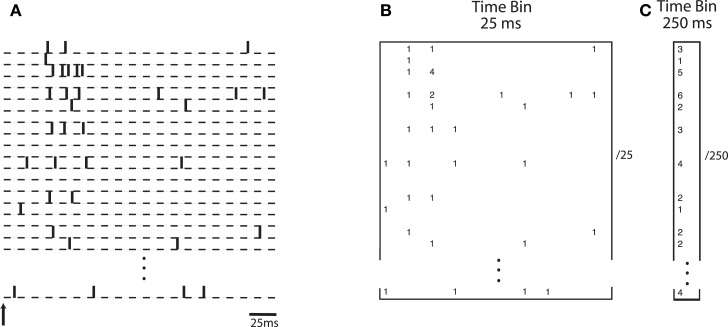
**(A)** Spike trains from simulated neurons are aligned relative to stimulus onset. Words are constructed by binning spike trains using a given temporal bin width. **(B)** A spatio-temporal word represented as a matrix of size *N* neurons by 10 time bins, each time bin being 25 ms wide. Numbers indicate spike counts for bins with at least one spike. **(C)** A word represented as a vector of length *N*, computed using a single bin of size 250 ms.

Specifically, we investigated whether the transient nature of the neurodynamics is critical for learning a linear discrimination boundary. If discrimination is driven by a sustained activation of neurons over time, then an optimal decoding strategy would be based on integrating the neuronal spiking activities over a substantial fraction of the trial. On the other hand, if the transient dynamics are critical for discrimination, then a finer grain temporal integration of the dynamics would yield better discrimination. We evaluated the difference between these two cases by constructing a neural “word” using integration time bins of different lengths. A time bin of 25 ms captures the transient information in the neural activities while a time bin of 250 ms would treat the neural information in a sustained manner. Theoretical studies have investigated how information content in the neural activity can vary as a function of the length of the window used for estimation (Panzeri et al., 1999). Here we empirically study this within the context of the neural activity generated by the model. Figure 6 illustrates how the spike count matrix for discrimination is constructed. Note that the dynamic word has a dimensionality that is 10 times larger than the sustained word.

**Figure 7:**
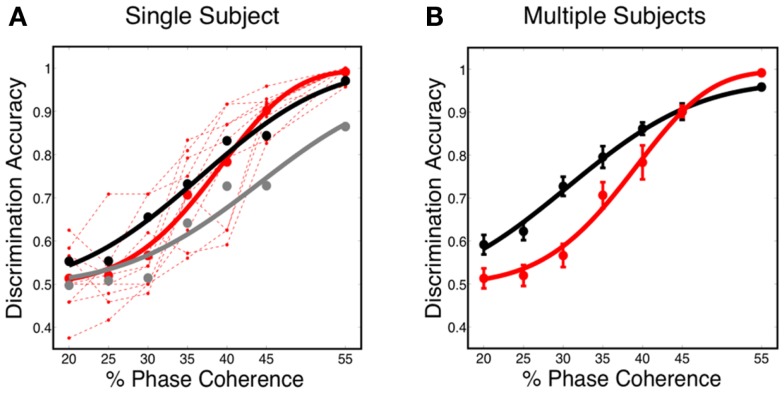
**(A)** Simulated neurometric performance for one initialization of the V1 model. Shown are (black) neurometric curve constructed by decoding the full spatio-temporal word, *D* = 3.19, *p* = 0.53; (gray) neurometric curve constructed from a decoder that ignores dynamics, *D* = 52.26, *p* < 0.01. Also shown is (dashed red lines) the psychophysical performance of 10 human subjects, and (solid red curve) the group psychometric curve across 10 subjects. **(B)** Shown are (black) simulated neurometric performance averaged over five initializations of the model, together with (red) the average psychometric curve for the 10 human subjects. Error bars on both curves represent standard error. Likelihood ratio test yields *D* = 6.42, *p* = 0.17.

The decoding results, shown in Figure 7A, reveal that the predicted discrimination accuracy is substantially higher when transient neurodynamics are utilized. The neurometric curve for the transient dynamics decoding (i.e., temporal coding model, black curve) lies within the variation of the human behavioral data, whereas the neurometric curve for the sustained decoding (gray curve) is clearly outside the range of human behavioral performance. Figure 7B shows the mean neurometric curve (black curve), constructed by averaging the discrimination accuracy across six random initializations of the model. The average psychometric curve (average of 10 subjects, red curve) is shown for comparison. The significance of the similarity between the neurometric and psychometric curves should not be overlooked, given that the model of V1 is not optimized/tuned to match the psychophysics nor is the decoder trained to match the psychophysics. Taken together with the fact that the V1 model also produces realistic classical and extraclassical physiological responses, at both the single cell and population level (Wielaard and Sajda, 2006a,b), this result provides validation for this modeling substrate.

**Figure 8:**
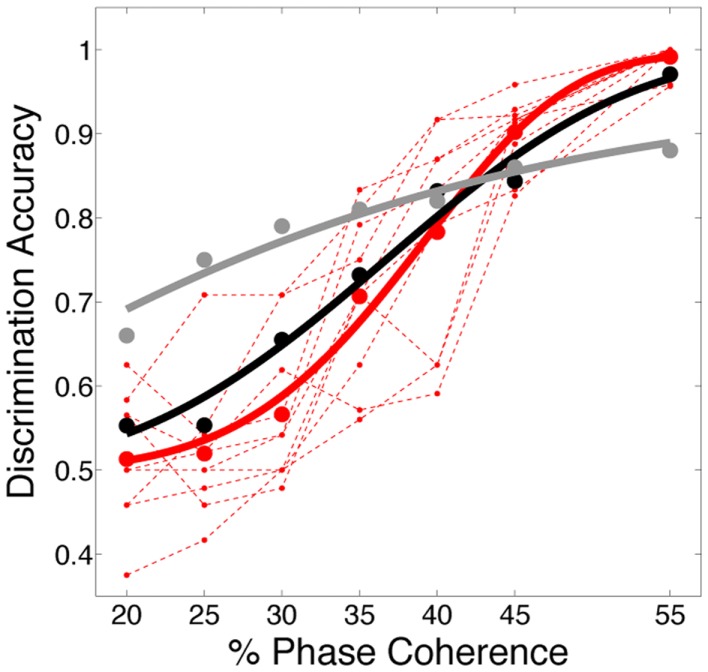
**Decoding accuracy compared to a feedforward model**. Comparison of psychometric and two neurometric curves, one for sparse linear decoding of activity generated by our dynamical V1 model (black curved – same as Figure 7A, *D* = 3.19, *p* = 0.53;) the other for a sparse linear decoding of activity generated by the first layer of the feed forward HMAX model (gray curve; *D* = 48.26 (*p* < 0.01). Code for simulating HMAX feedforward model is freely available from http://cbcl.mit.edu/jmutch/cns/hmax/doc/

We further investigated the role dynamics plays in decoding accuracy and the fit of the neurometric curve relative to human psychometric performance by comparing the decoding of activity generated by our V1 model to the activity generated by the first layer of the feedforward HMAX model (Serre et al., 2007). Shown in Figure 8, this version of HMAX, which has no dynamics, results in a neurometric curve that substantially deviates from the psychometric performance, overestimating decoding accuracy at low coherence levels and underestimating it at high coherence levels.

Finally we investigated how decoding accuracy is affected by the degree of sparsity in the neural activity. Table 1 was constructed by varying the initial conditions of the model in such a way that physiological response properties, such as orientation tuning and modulation ratios (i.e., fraction of simple and complex cells), were not significantly affected though there were observed changes in both spatial and temporal sparsity. As can be seen in Table 1, the decoding accuracy is strongly correlated with the spatial sparseness of the activity (Pearson’s *r* = 0.96, *p* = 0.009) but not significantly correlated with temporal sparseness (Pearson’s *r* = −0.65, *p* = 0.24) with the higher decoding accuracy associated with a greater degree of spatial sparseness.

**Table 1:** **Five V1 models differing in their spatial and temporal sparsity, measured via kurtosis, and the resulting classification performance measured via the area under the ROC curve (Az)**.

	Spatial Sparseness (Kurtosis)	Temporal Sparseness (Kurtosis)	Classification (Az)
Model 1	8.50	2.51	0.73
Model 2	11.83	1.42	0.84
Model 3	14.91	0.08	0.87
Model 4	8.84	0.60	0.76
Model 5	9.86	1.15	0.78

### Alternative decoding strategies

**Figure 9:**
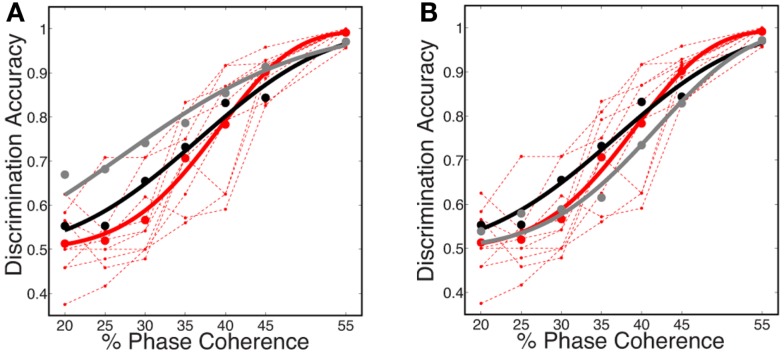
**Comparison of several decoding strategies**. **(A)** Two decoding strategies: (black) train and test at each coherence independently, *D* = 3.19, *P* = 0.53; (gray) train at the highest coherence and test at each coherence, *D* = 12.88, *P* = 0.01. Both are trained as sparse decoders. **(B)** Sparse and non-sparse decoding strategies: (black) sparse decoder, *D* = 3.19, *P* = 0.53; (gray) non-sparse decoder each with the same amount of regularization, *D* = 3.66, *P* = 0.45. Both are trained on each coherence independently. Also shown are (dashed red lines) the psychophysical performance of 10 human subjects; (solid red curve) the group psychometric curve across 10 subjects.

In this work, we use the decoder to evaluate the information content for varying level of stimulus noise. In this regard, we compared several decoding strategies. Figure 9A shows neurometric curves for two decoding scenarios: (black) train and test the decoder at each coherence independently (gray), train at the highest coherence and test at each coherence. Simulation results indicate training and testing the decoder on each coherence level independently, though suboptimal relative to training at the highest coherence, is a better match to the human psychophysics. This would suggest that not only information about the signal but also information about the noise distribution is potentially used by the subjects in the task.

Figure 9B compares sparse and non-sparse decoding strategies: (black) sparse decoding (gray), non-sparse decoding. In this case it is less clear which is a better match to the human psychophysics – i.e., both lie within the inter-subject variability and both are fits cannot be rejected under the likelihood ratio test (See Statistical Tests). However, below and in the following sections we will discuss several advantages of the sparse decoder in terms of robustness.

**Figure 10:**
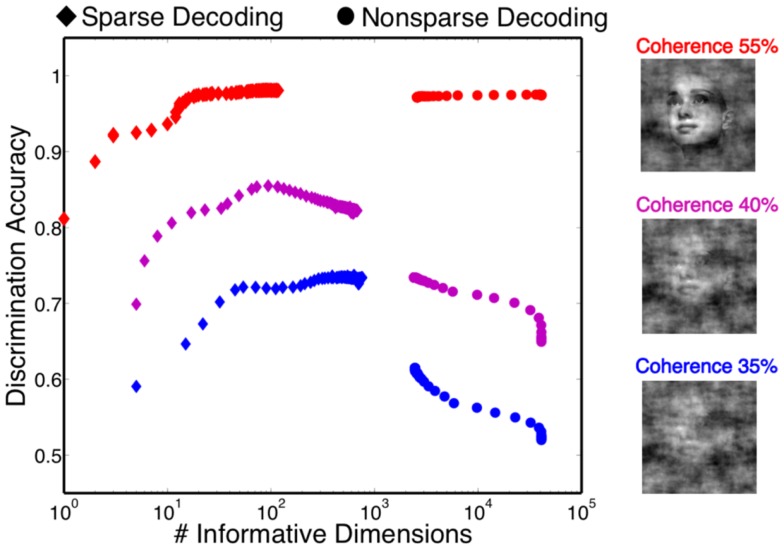
**Discrimination accuracy for both sparse and non-sparse decoding strategies**. Decoding is shown for three different coherence levels while sweeping the hyperparameter that controls the amount of regularization. Sparse decoding always yields fewer informative dimensions while also having greater discrimination accuracy for each of the three coherence levels.

One of the attractive features of a sparsity constraint is it makes the decoder robust to noise and irrelevant features (Ng, 2004). We tested robustness by comparing the sparse decoder with a non-sparse decoder (see Materials and Methods). Figure 9 compares the decoding results for the two different regularizations. We swept the values for the hyperparameters weighting the contribution of the regularizer (see Materials and Methods) and computed a cross-validated discrimination performance. Consistent with Figure 9B, the sparse decoder yielded better discrimination performance for all coherence levels, while also requiring one to two orders of magnitude fewer informative dimensions. Also clear from Figure 10 is a degradation of discrimination performance using non-sparse decoding, with such degradation more dramatic as the noise in the stimulus increases.

In general, sparsity helps in two ways: one is when the training samples are few, the other is its ability to ignore noise. The computer vision and deep learning community have investigated the importance of sparsity in classification tasks, with sparse autoencoding being an example of how sparsity can improve the final classification result (Ranzato et al., 2007; Coates and Ng, 2011).

### Informative dimensions

**Figure 11:**
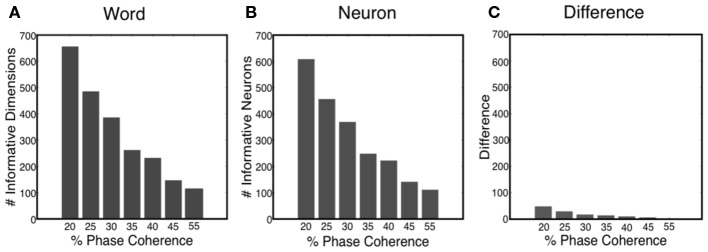
**(A)** Number of informative dimensions decrease as the task becomes easier. **(B)** Number of informative neurons decreases as the task becomes easier. **(C)** Difference between the number of informative dimensions and the number of informative neurons.

The sparse decoder not only makes decoding of high-dimensional features feasible, but also facilitates an avenue for investigating feature selection and quantifying the informative dimensions. Figure 11A shows that the number of informative dimensions increases as the task becomes more difficult, i.e., the decoder will exploit additional dimensionality in the neural word to improve decoding performance. Realizing the fact that one neuron might participate in the decision process multiple times, we also computed the number of informative neurons, which is the number of uniquely chosen neurons in the neural word. Our results indicated that the number of informative neurons also decreases as the task gets easier, as shown in Figure 11B. Such an observation offers possible insight into the tradeoff between decoding accuracy and metabolic cost. Finally, Figure 11C shows the number of neurons in which more than one time bin is selected in the neural word. Thus most neurons participate only once in the decoding process.

### “Melody” within neural dynamics

**Figure 12:**
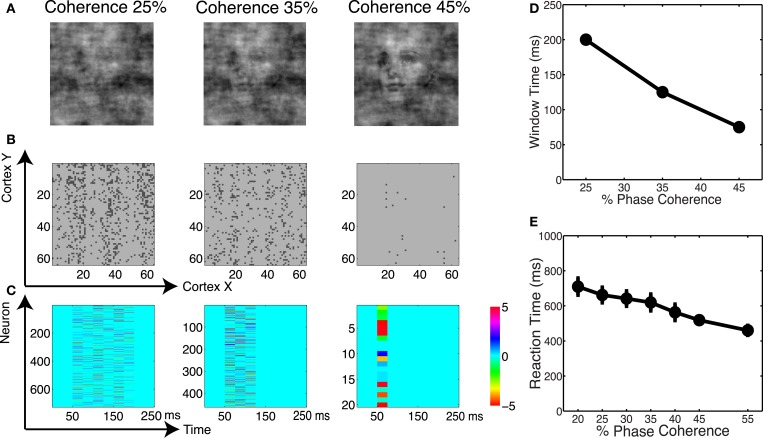
**(A)** Example of images at three coherence levels. **(B)** Spatial distribution of neurons selected by the decoder. Shown are neurons in the cortical space, where selected neurons are indicated in black. **(C)** Temporal windows selected by the decoder together with their corresponding weights. **(D)** Chronometric function indicating the time needed by the decoder to accumulate evidence in the network dynamics. **(E)** Behavioral chronometric functions derived from human psychophysics.

We analyzed the spatial and temporal distributions of the informative dimensions arising from the sparse decoding framework. Figure 12A shows three coherence levels while Figure 12B shows the spatial distribution of neurons selected for these three levels. It is clear that more neurons are selected as the task becomes more difficult. Given that the stimuli are presented as a monocular simulation, one can see “banding” indicative of ocular dominance columns. We also investigated the temporal distribution of the selected neurons. Using an adaptive windowing technique (described in the Materials and Methods) we found a pattern of activity that can best be described as a “melody.” See Figure 12C, which shows that the visual stimulus elicits a spatio-temporal pattern in the cortical activity that carries information about discrimination. Our finding is similar to the activation patterns that have been observed *in vitro* for spontaneous cortical activity (Cossart et al., 2003; Watson et al., 2008).

The adaptive windowing technique also allows us to investigate the optimal time window over which the decoder integrates information to form a decision. Figure 12D can be interpreted as the network’s chronometric function, namely the time needed for the network to form the decision. Though the network does not consider motor preparation or other possible sources of delay, and thus the chronometric function cannot be strictly interpreted as a measure of reaction time, this trend is very consistent with subjects’ reaction time curves, see Figure 12E. Thus we see that by reading out the neurodynamics of the V1 model using a sparse linear decoder, we observe accuracy and timing for the discrimination that are very much consistent with the results for human subjects.

## Discussion

### Decoding spatio-temporal dynamics

Experimental studies, both on the microscopic and macroscopic level, have offered insight that higher areas of cortex convey adequate neural evidence for decision making, whereas the biological transformation of such evidence through the early visual pathway remains unclear. Our results suggest that there is substantial information at the level of V1 that can be linearly decoded and mapped to behavior data. A study of optical imaging data in awake behaving monkeys investigated the decoding capacity of V1 and showed that an optimal decoder could explain the behavioral response in a reaction time visual detection task (Chen et al., 2006, 2008). A follow-up study using single-unit and multi-unit recordings in the same task showed an inferior decoding performance for V1 (Palmer et al., 2007), indicating that pooling from a large neuronal population is critical for exploiting the maximum information encoded in the cortex. Our work suggests that this pooling must be done in a distributed manner across the population, namely simply locally pooling neurons in the population does not suffice. We also find that short sampling intervals/windows (25 ms) yield better discrimination accuracy.

As we mentioned earlier, a more recent primate study has shown how an empirically based linear decoder can be used to decode firing rate activity from a population of V1 neurons (on the order of 100 neurons) for a 2-AFC orientation discrimination task (Graf et al., 2011). These results also showed that specific subsets of neurons in the population contribute to the decoding accuracy.

Our results also support the hypothesis of DiCarlo et al. who have argued that the decision space for invariant object recognition is a subspace which flattens out non-linear manifolds of the early visual representation (DiCarlo and Cox, 2007). Our results agree with this hypothesis and suggest that even at the level of V1, substantial information can be decoded using a linear decoder. It should be noted that our work has been mostly focused on evaluating invariance along the dimension of spatial noise, with stimuli normalized in position and scale.

### Relationship to other decoding methods

Several investigators have argued for population coding (Pouget et al., 2000, 2002; Averbeck et al., 2006) as a model of orientation discrimination, based on the assumption that orientation tuning, computed from an average response over the period of sinusoidal drifting gratings, was responsible for discrimination. Models based on selecting the most informative point on a tuning curve, usually the steepest point, only utilized the local shape of the tuning curve (Bradley et al., 1987; Hawken and Parker, 1990). Fisher information was utilized in later models to study the discriminating information for tuning curves (Seung and Sompolinsky, 1993; Abbott and Dayan, 1999; Sompolinsky et al., 2001) under the assumption that information was not limited to a point on the tuning curve, but was subject to the limitation that Fisher information could only be measured for a very small orientation difference. Besides the population code, a further study using the Chernoff distance between two distributions of spike counts paid attention to the global information of a tuning curve, and found that narrow orientation tuning was not necessarily optimal for all angular discrimination tasks (Kang and Sompolinsky, 2001; Kang et al., 2004). These studies, however, all resorted to using the average responses of neurons across time, therefore ignoring the rich dynamics of the spiking activity of the neuronal population.

On the other hand, substantial work has been done to characterize the temporal characteristics of neural dynamics using statistical models. Bayesian methods (Bialek and Zee, 1990; Bialek et al., 1991; Sanger, 1996; Osborne et al., 2005) are used to characterize the spiking dynamics of neurons, providing the flexibility for statistical inference (Mendel, 1995; Brown et al., 1998). Point process likelihood-based generalized linear models (GLM) have been used to analyze history dependence in neural spiking activity. State-space models provide a flexible tool for neural spike train decoding (Brown et al., 1998; Barbieri et al., 2004; Brockwell et al., 2004; Paninski, 2004; Wu et al., 2006; Deneve et al., 2007; Czanner et al., 2008). These statistical methods typically model the neural dynamics with some assumptions on the hidden brain states, and use a generative approach to infer the stimuli based on the neural dynamics. Latency coding scheme (Shriki et al., 2012) and spike timing dependent plasticity (Masquelier and Thorpe, 2007) have been investigated for decoding V1 information, all pointing to the conclusion temporal dynamics is critical for object recognition. Our results have focused on a discriminative approach, mapping the neural dynamics into a (binary) decision.

### Relationship to reservoir computing

Theorists have sought to build computational models that are inspired by the cortex and test how such architectures perform in object recognition tasks. Models based on feedforward architectures, such as HMAX (Serre et al., 2007), propose that a combination of linear and non-linear filtering operations, arranged in a hierarchical structure, can map the visual world into a high-dimensional space where objects can be linearly decoded with high recognition accuracy. Noticeably absent in such feedforward architectures is a recurrent connectivity and thus the dynamics which are clearly prevalent in visual cortex. Termed liquid state machine (LSM) (Maass et al., 2002) and echo state network (ESN) (Jaeger and Haas, 2004), these algorithms have been related to the brain’s neural microcircuitry, and potentially link the anatomy and physiology of the vast recurrent circuitry in cortex with general computation capabilities.

The concept of reservoir computing stipulates the importance of dynamics. Theoretically, there are several ways to utilize the dynamics of the population spike trains. The first approach is to study the spike statistics, such as mutual information, though such an approach is limited to a small number of neurons. A second way of using dynamics relies on cliques of neurons falling into a stable state, such as a limit cycle, as a model of coding information and short-term memory. LSM and ESN, however, do not rely on forming stable brain states, as a traditional dynamical system would require. A number of investigators have pursued work along this line, most of which use randomly connected recurrent networks (White et al., 2004; Yamazaki and Tanaka, 2007; Legenstein et al., 2008). Our work follows this line of research, however using a physiologically realistic model of V1 that was constructed under many biological constraints.

### Caveats of a model

A potential criticism of this work is that our results are based on decoding of activity generated from a computational model of V1 and not from experimental recordings. Our model was designed to investigate classical and extraclassical response properties of large population of neurons in early visual cortex. Thus though the neural activity we decode is simulated, our model is physiologically realistic, validated against experimental data across a wide range of responses.

Complex visual objects are likely not directly discriminated from V1 responses alone, and more complex non-linearities and dynamics, including feedback, likely play a role in how objects are detected, identified, and recognized. However we believe that the paradigm presented in this paper represents a first step in understanding how complex images might be encoded within the V1 activity and be ultimately decoded further downstream.

## Conflict of Interest Statement

The authors declare that the research was conducted in the absence of any commercial or financial relationships that could be construed as a potential conflict of interest.
